# CONGENITAL HYPOTHYROIDISM AS A RISK FACTOR FOR CENTRAL HEARING PROCESS DISORDERS

**DOI:** 10.1590/1984-0462/;2019;37;1;00014

**Published:** 2018-08-30

**Authors:** Caio Leônidas Oliveira de Andrade, Aline Cupertino Lemos, Gabriela Carvalho Machado, Luciene da Cruz Fernandes, Lais Luz Silva, Hélida Braga de Oliveira, Helton Estrela Ramos, Crésio Aragão Dantas Alves

**Affiliations:** a Universidade Federal da Bahia, Salvador, BA, Brasil.; b União Metropolitana de Educação e Cultura, Lauro de Freitas, BA, Brasil.

**Keywords:** Central auditory diseases, Neonatal screening, Congenital hypothyroidism, Cognition, Doenças auditivas centrais, Triagem neonatal, Hipotireoidismo congênito e cognição

## Abstract

**Objective:**

To investigate the presence of central auditory processing disorder symptoms in children with congenital hypothyroidism.

**Methods:**

An exploratory, descriptive, cross-sectional study of 112 patients with congenital hypothyroidism aged ≥5 years old. An interview was held with the parents/caregivers at the time of the medical consultation. Patients with other medical conditions were excluded. As a research instrument, the structured protocol of anamnesis was used to evaluate the auditory processing routinely used by audiologists. For statistical analysis, the chi-square test was used.

**Results:**

Sex distribution was similar in both boys and girls (girls: 53.3%). The most prevalent phenotypic form of congenital hypothyroidism was no dysgenesis (88.4%), and 65.3% of the children had an episode of irregular serum thyroid-stimulating hormone (TSH) levels. Among the manifestations of the most frequent central auditory processing disorder symptoms, problems were reported with regard to cognitive functions, as they related to hearing, such as figure-background ability (83.0%), auditory attention (75.9%) and auditory memory (33.0%). Complaints related to school performance were reported in 62.3% of the cases.

**Conclusions:**

The data obtained show a high frequency of lag symptoms in cognitive functions related to central auditory processing, particularly with regard to auditory attention, figure-background ability and auditory memory in patients with congenital hypothyroidism.

## RESUMO

**Objetivo:**

Investigar a manifestação de sintomas do transtorno do processamento auditivo central em crianças com hipotireoidismo congênito.

**Métodos:**

Estudo de caráter exploratório, descritivo e transversal com 112 pacientes com hipotireoidismo congênito com idade ≥5 anos. Realizou-se entrevista com os pais/cuidadores no momento da espera da consulta médica. Portadores de outras afecções médicas foram excluídos. Como instrumento de pesquisa utilizou-se o protocolo estruturado de anamnese para avaliação do processamento auditivo rotineiramente empregado por audiologistas. A análise estatística utilizou o teste Qui-quadrado.

**Resultados:**

A distribuição por sexo foi semelhante (meninas: 53,3%). Os casos não-disgenesia constituíram a forma fenotípica mais prevalente para o hipotireoidismo congênito (88,4%), e verificou-se que 65,3% das crianças apresentavam algum episódio de níveis séricos irregulares de hormônio tireoestimulante. Dentre as manifestações mais frequentes dos sintomas do transtorno do processamento auditivo central, as queixas relaciondas às funções cognitivas auditivas, como: figura-fundo (83,0%), atenção auditiva (75,9%) e memória auditiva (33,0%) foram as mais evidentes. Reclamações relacionadas ao rendimento escolar foram reportadas em 62,3%.

**Conclusões:**

Os dados obtidos evidenciaram altas frequências de sintomas de defasagem nas funções cognitivas relacionadas ao processamento auditivo central, em especial na atenção auditiva, figura-fundo e memória auditiva nos portadores do hipotireoidismo congênito.

## INTRODUCTION

Congenital hypothyroidism (CH) is one of the most common endrocrinopathlogies in childhood, with a worldwide incidence of 1:3000/4000 births.^1^ It is characterized by the absence or decrease in thyroid hormones (THs) and may occur in two common clinical forms: dysormonogenesis (10-15%) and dysgenesis (80-85%).^2^

Studies have shown that 5% of thyroid dysgenesis (TD) cases are associated with mutations in genes responsible for the growth or development of thyroid follicular cells. This occurs due to a large variety of different structural malformations in the thyroid that result in a wide range of different CH phenotypes, (e.g.: NKX2.1 e FOXE1, PAX8 e TSHR), displaying an extremely complex pathogeny.^3^ ^,^ ^4^ The dysgenetic group contains agenesis (or hemigenesis), hypoplasia, and the ectopic gland. Conversely, dyshormonogenesis occurs when there are autosomal recessive mutations of key molecules regulating thyroid hormone synthesis.^5^

Irregular or insufficient TH intake in early stages of gestation and in early years of life has been related to neural connective damages.^6^ The triiodothyronine (T3) hormone is essential for the maturation of complex brain function and for somatic growth.^7^ THs are essential for metabolic development, growth, homeostasis, and for the morphophysiological maturation of central auditory pathways.^8^ ^,^ ^9^

After understanding the role of THs in the auditory system, it is plausible to suppose that a lack of these hormones may cause alterations in the processing of the acoustic signal from the peripheral pathways to the auditory cortex, which are expressed as linguistic, cognitive, school performance, and/or socioemotional problems, thus characterizing central auditory processing disorders (CAPD) and their manifestations.

The Central Auditory Process (CAP) is the physiological mechanism for conducting auditory information from the cochlea, the peripheral sensory organ, to the upper auditory centers.^10^ In order to enable adequate processing of these physiological functions, the integrity of these pathways is essential. These pathways allow individuals to use their auditory abilities, such as detecting, discriminating, recognizing and understanding (all acoustic signal information).^11^ Furthermore, it enables them to communicate and organize themselves in the spaces in which they live.

Appropriate treatment of CH at early ages is essential to prevent and/or minimize damage to the pathways responsible for central auditory processing. When children’s disorders are detected, therapeutic measures should be adopted as early as possible in order to minimize deleterious effects.

There is a lack of studies regarding CAPD in CH subjects because the majority of research projects have prioritized understanding audiometric thresholds, and to a lesser extent, electroacoustic and electrophysiological hearing processes, rather than investigating the impairment of cognitive functions related to hearing. In view of this, the aim of this study was to investigate the presence of signs and symptoms of auditory processing disorders in children with CH, and to verify the association of this disorder with their clinical and laboratory aspects, with the purpose of demonstrating the relevance of evaluating central auditory processing in children/the population of this age group.

## METHOD

We evaluated 112 patients diagnosed with congenital hypothyroidism aged ≥5 years-old (range 5-16) of both genders, who were treated at the Neonatal Screening Service in the State of Bahia (Northeast Brazil) in the year 2014, with the following exclusion criteria: subjects with syndromes (Down Syndrome, Pendred Syndrome, Kabuki Syndrome, etc.), neurological diseases or psychiatric disorders, which were diagnosed by using information collected directly from medical records; those with a history of middle and/or external ear diseases; those presenting risk factors for hearing loss, or reporting current or past infectious diseases involving the central nervous system (CNS); subjects with other metabolic diseases and any other form of hypothyroidism that was not permanent congenital hypothyroidism.

This is an exploratory, descriptive and cross-sectional study, with a convenience sample, obtained by evaluating all patients with CH from March to October 2014. The project was approved by the Ethics Committee (Opinion No. 534,704). All of the participants volunteered to participate in the research. A Free and Informed Consent Form was signed by the patients’ parents or guardians, and the patients signed the Free and Clarified Consent Form, whenever appropriate.

In this analysis, the parents or guardians were the source of information consulted to identify the presence of auditory symptoms. Three research instruments were used: the first, for a preliminary investigation about the presence of risk factors for hearing loss and anamnesis - a history of other diseases and family history of hearing loss; the second, routinely applied by audiologists^12^ to collect data regarding the symptoms of central auditory processing disorders and their implications on the child’s school, emotional and social life. The last instrument was a structured questionnaire composed of 25 closed and open-end questions, divided into two sections. The first section consisted of 10 closed-ended, dichotomous (Yes or No) questions that directed the investigation with regard to the presence of specific symptoms of the CAPD. The second section had 15 open-ended and closed-ended (Yes or No) questions, and investigated signs of co-morbidities associated with CAPD, such as school-related and social difficulties.

The parents/caregivers were submitted to a formal interview process. A “face to face” technique was used in which the researcher/interviewer read the items of the questionnaire to the interviewee and, after providing appropriate instructions, did not interfere farther in the process.

The clinical and laboratory data of the patients were extracted from medical records. The severity of CH was classified based on serum levels of total T4 (≥ or <2.50 µg/dL) at the time of the diagnostic examination. The individuals who had three or more episodes of serum thyroid-stimulating hormone (TSH) levels <0.5 µUI/mL or >15 µUI/mL were considered to be patients with irregular hormonal serum levels, and were classified as hypertreated and hypotreated patients, respectively, for the purpose of establishing an association between hormonal control and central auditory processing manifestations.

The etiology of CH was classified according to ultrasonography, and when necessary, by thyroid scintigraphy into: dysgenesis and no dysgenesis. The cases of dysmormonogenis could not be identified by the perchlorate test, which showed evidence of partial iodide organification defects, thus it was difficult to classify cases with topical thyroid glands and the absence of TH as dysmormonogenesis.

For statistical analysis, SPSS software, IBM Corporation, Chicago, USA (version 21.0) was used. The continuous variables were described by mean values, standard deviation, relative frequencies and strength of association. The bivariate analyses between categorical variables were performed with the chi-square test, at a significance level of 5% (p<0.05).

## RESULTS

The mean age and the time since the diagnosis of the disease among the individuals with CH was 8.6 years (±2.9). The sample predominantly consisted of girls (53.3%). Among the etiological factors, the most prevalent phenotypic form (88.4%) was found in the cases of no diysgenesis. In the hormonal follow-up, 65.3% of the individuals had at least one episode of irregular serum TSH levels. Of these, 48.2% of the subjects had episodes of TSH suppression (≤0.5 µUl/mL) and 17.1% had increased TSH levels (≥15 µUI/mL). Only 14.3% demonstrated regular serum TSH levels throughout the treatment.

**Figure 1:**
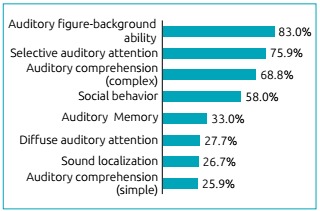
Distribution of the relative frequency of auditory processing disorder symptoms in the different hearing-related cognitive functions in individuals affected by congenital hypothyroidism (n=112).

**Figure 2:**
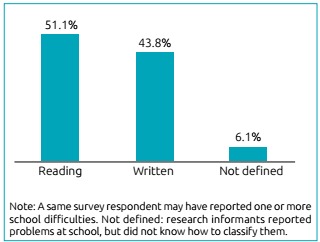
Relative frequency of difficulties at school in individuals affected by congenital hypothyroidism (n=112).

Among the manifestations of CAPD present in the sample, as shown in Figure 1, the cognitive functions of auditory figure-background ability (83.0%), selective auditory attention (75.9%), and auditory comprehension of complex orders (68.8%) were the symptoms most frequently mentioned by parents or guardians. There were also reports related to school problems, with 62.3% of the families reporting one or more complaints related to their child’s learning. Among the complaints, difficulties in reading (51.1%) and writing (43.8%) were the most frequent (Figure 2).

Tables 1 and 2 show the manifestations of CAPD in individuals with CH, and the strength of associations with clinical, phenotypic and laboratory variables. It was possible to verify that the patient’s age at the onset of treatment, disease severity, and etiology of the CH were identified as having significant associations, with reports of difficulty in understanding simple orders, selective auditory attention and auditory figure-background ability.

**Table 1:** Strength of association between clinical-laboratory variables and the main symptoms of auditory processing disorders (communicative and social behavior) in the participants of the study (n=112).

Clinical and laboratory findings	Main manifestations of auditory processing disorders (communicative and social behavior)							
	Auditory comprehension of simple orders		Sound localization		Social behavior		Auditory comprehension of complex orders	
	%	PR (95%CI)	%	PR (95%CI)	%	PR (95%CI)	%	PR (95%CI)
Time of disease diagnosis/treatment (days)								
≤7	20.5	1	30.8	1	69.4	1	69.2	1
>7	31.9	1.55 (0.74-3.28)	27.7	0.89 (0.46-1.74)	63.8	0.92 (0.68-1.25)	63.8	0.92 (0.68-1.24)
p-value	0.232		0.132		0.845		0.086	
Age - Onset of treatment (days)								
≤28	25.0	1	25.0	1	87.5	1	50.0	1
>28	25.0	1.00 (0.28-3.54)	26.4	1.05 (0.30-3.72)	61.4	0.70 (0.51-0.97)	70.8	1.41(0.70-2.88)
p-value	0.986		0.946		0.335		0.489	
Severity of disease (T4 Total neo)								
>2.5 µg/dL	37.3	1	27.5	1	60.8	1	72.5	1
<2.5 µg/dL	8.3	0.22 (0.03-1.51)	36.4	1.32 (0.54-3.26)	58.3	0.96 (0.57-1.62)	72.7	1.00 (0.67-1.49)
p-value	0.024		0.555		0.745		0.990	
Treatment condition								
Normotrated (0.5-15 µUI/mL)	25.0	1	18.8	1	66.7	1	75.0	1
Hypertreated (>0.5 µUI/mL)	29.6	1.18 (0.46-3.04)	27.8	1.48 (0.49-4.48)	62.3	0.93 (0.62-1.41)	63.0	0.83 (0.59-1.19)
Hypotreated (>15 µUI/mL)	15.8	0.62 (0.17-2.41)	36.8	1.96 (0.61-6.38)	72.2	1.08 (0.68-1.71)	73.7	0.98 (0.67-1.45)
p-value	0.788		0.371		0.818		0.274	
Etiology of CH								
No Dysgenesis	28.3	1	26.3	1	59.4	1	69.7	1
Dysgenesis	9.1	0.31 (0.05-2.14)	36.4	1.38 (0.59-3.23)	72.7	1.22 (0.82-1.82	72.7	1.04 (0.71-1.53)
p-value	0.027		0.833		0.322		0.927	

PR: Prevalence Ratio; CI: Confidence Interval; Neo: neonatal; CH: Congenital hypothireoidism.

**Table 2:** Association between clinical-laboratory variables and the main symptoms of auditory processing disorders (cognitive subprocesses) in the participants of the study (n=112).

Clinical and laboratory findings	Main manifestations of auditory processing disorders (cognitive subprocesses)							
	Diffuse auditory attention		Auditory memory		Selective auditory attention		Auditory figure-background ability	
	%	PR (95%CI)	%	PR (95%CI)	%	PR (95%CI)	%	PR (95%CI)
Time of disease diagnosis/treatment (days)								
≤ 7	28.2	1	31.4	1	79,5	1	86,1	1
>7	25.5	0.90 (0.45-1.82)	34.0	1.08 (0.58-2.04)	74,5	0.93 (0.74 -1.18)	85.1	1.01 (0.83-1.18)
p-value	0.187		0.500		0.273		0.596	
Age - Onset of treatment (days)								
≤28	12.5	1	25.0	1	37,5	1	62.5	1
>28	26.4	2.10 (0.32-13.8)	40.6	1.62 (0.47-5.57)	81,9	2.18 (0.89-5.38)	88.6	1.41 (0.82-2.44)
p-value	0.782		0.634		0.003		0.050	
Severity of disease (T4 Total neo)								
>2.5 µg/dL	23.5	1	34.0	1	80,4	1	84.6	1
<2.5 µg/dL	36.4	1.54 (0.61-3.90)	41.7	1.22 (0.57-2.65)	100	1.24 (1.09-1.42)	100	1.18 (1.05-1.33)
p-value	0.625		0.799		0.126		0.379	
Treatment condition								
Normotrated (0.5-15 µUI/mL)	18.8	1	21.4	1	68,8	1	87.0	1
Hypertreated (> 0.5 µUI/mL)	33.3	1.78 (0.60-5.27)	39.6	1.85 (0.64-5.32)	75,9	1.10 (0.77-1.59)	82.7	0.95 (0.75-1.21)
Hypotreated (> 15 µUI/mL)	21.1	1.12 (0.29-4.29)	38.9	1.81 (0.57-5.78)	84,2	1.22 (0.83-1.80)	95.0	1.09
p-value	0.521		0.669		0.886		0.645	
Etiology of CH								
No dysgenesis	28.3	1	31.6	1	78,8	1	85.6	1
Dysgenesis	27.3	0.96 (0.35-2.66)	63.6	2.01 (1.18-3.44)	63,6	0.80 (0.51-1.28)	100	1.16 (1.08-1.27)
p-value	0.927		0.106		0.010		0.436	

PR: Prevalence Ratio; CI: Confidence interval; Neo: neonatal; CH: Congenital hypothireoidism.

Tables 1 and 2 show that the highest prevalence of symptoms reported were also centered on the cognitive abilities of selective auditory attention and auditory figure-background ability, the latter having a strong association with all of the clinical, phenotypic, and laboratory variables analyzed. Although there was a strong association between the irregular treatment of CH with almost all of the CAPD-related symptoms reported, it is worth mentioning that hypotreatment was the most evident among the associations.

## DISCUSSION

The present study investigated the presence of CAPD manifestations in individuals with HC by collecting quantitative information on signs and symptoms that showed impairments in auditory-related cognitive functions. These were more relevant in children whose clinical and laboratory variables were in disagreement and who had a good prognosis of the treatment of the disease.

Although it has been extensively discussed in the literature, the pathogenesis of CAPD is not yet well-elucidated. Nevertheless, the symptoms and mechanisms of CAPD are well-known and well-described, and one or more alterations in hearing-related cognitive functions have been shown to suggest this disorder.^13^ When these alterations occur at an early age, even if they are subtle, they may compromise the development of language and learning.^14^ ^,^ ^15^

In this study, the magnitude of the prevalence of symptoms associated with CAPD in CH was associated with factors such as: age over 28 days at the onset of treatment, severity of hormone deficiency, disease etiology, treatment condition during hormonal follow-up, and to a lesser extent, the time since diagnosis of the disease, and time of the disease treatment, which was different from some studies.^16^ ^,^ ^17^ This difference may be related to the mean age of the sample, which was lower than that in most of the other similar studies. In the literature, these factors are related to the severity of changes in the overall development of this population,^17^ ^,^ ^18^ especially when the onset of treatment is delayed, and/or hormone deficiency is more severe, causing deleterious effects on cognitive and motor functions.^16^ ^,^ ^17^

In addition, the etiology of CH and the conditions of treatment have a direct influence on language alterations^19^ ^,^ ^20^ and hearing.^21^ Particularly, regarding the etiological factor, a higher prevalence of no dysgenesis was observed. These were probably cases of dysormonogenesis. In the literature, most cases of CH (85%) are related to dysgenesis.^2^ This difference could be related to the fact that it was not possible to identify the cases of dysormonogenesis using the perchlorate test, since the institution where the research was conducted does not perform this procedure. However, in the last two decades, studies^22^ have shown an increased frequency of CH diagnosis with the thyroid gland in situ, probably due to the earlier detection and onset of treatment. This is associated with a higher dose of levothyroxine sodium (T-L4), resulting in the early normalization of serum levels of TH in screening programs. Thus, there is evidence that dysormonogenesis (no dysgenesis), a less severe form of the disease, is associated with a higher risk and severity of hearing loss,^21^ ^,^ ^23^ but thyroid agenesis is more directly related to language disorders^24^, probably because it has more significant influence on the cognitive functions of central auditory processing, such as auditory memory abilities, sound localization, auditory figure-background ability, and auditory comprehension of complex orders, according to the prevalence of symptoms found and associated with this clinical aspect in the present study.

The aforementioned alterations are dependent on the timing of TH deficiency in the brain areas responsive to perceptual processes, and may be primary to CAPD, such as auditory dysfunctions, or secondary to CAPD, such as language and cognitive alterations. These findings are in agreement with symptom associations related to cognitive function deficits involved in central auditory processing, with risk factors in CH, and with a higher frequency of symptoms related to cognitive figure-background ability, selective auditory attention, and auditory memory in the individuals that are the most exposed.

There were also significant records of signs and symptoms related to social and communicative behavioral aspects, with a positive association between the prevalence of those exposed to risk factors for CH, compared to non-exposed individuals, especially in patients with frequent episodes of hypotreatment. This finding is particularly important as it signals that the time periods when the TSH is below the cut-off point during treatment is cause for concern, because it is harmful to CNS. This is different than transient hyperthyroxinemia, which is usually common during treatment.^25^ Thus, it is imperative for all health professionals involved in the treatment of CH to be aware of and pay attention to hypotreatment in their patients.

As a result of the significantly high frequency of symptoms of cognitive function changes in the present study, complaints related to children’s academic performance were moderately prevalent in the sample, demonstrating that the CAPDs are related to the learning disorders^14^ that are common in children with CH.^20^ These findings suggested a delay in the maturation of the cognitive functions related to hearing in CH, and the school difficulties secondary to the lags in auditory processing - more specifically, the functions regarding figure-background ability, selective attention and auditory memory - were leading to problems in learning, especially in reading and writing.^26^

Some studies have reported an association between CH and attention and memory problems, contributing to specific learning disorders.^17^ ^,^ ^20^ Because attention is a multimodal process, it is important for school performance,^27^ and it is impaired when a child has difficulties concentrating on a target stimulus for a given period. In central auditory processing disorders, children show a change in directed attention, are easily distracted, and have difficulties understanding speech when there is noise in the background. Therefore, they often have low academic performances.^28^ Likewise, memory mechanism deficits interfere with academic performance. This type of cognitive function is essential in all learning processes, given its role in the storage of acquired information.^29^ The lack of or the decrease in TH has a negative influence on hippocampus functions and may be associated with changes in memory, since working memory is one of the most compromised functions.^30^ ^,^ ^31^

Furthermore, according to the results of the present study, damage to communicative aspects was shown in CH, as a consequence of the symptoms related to hearing difficulties and difficulties in comprehending complex orders. The literature reports that a change in hearing-related cognitive functions may lead to difficulty in understanding speech, because it interferes with the ability to decode phonemic aspects of speech.^32^ The communicative performance of individuals with CH is often altered,^18^ ^,^ ^24^ with a predominance of a delay in oral language acquisition, difficulties in understanding speech, phonetic and phonological deviations, the morphosyntactic structure, and lags in naming and acquiring vocabulary.^18^ ^,^ ^19^ ^,^ ^29^ ^,^ ^33^.

This study showed that CAPD may be present in children with CH, with a strong association between the presence of CAPD symptoms and clinical and laboratory indicators of variables related to a non-optimal prognosis of the disease. Thus, the study verified the relevance of evaluating and monitoring central auditory processing in this population.

The main limitations of this study were the use of a convenience sample and the lack of a control group. Other limitations were that no subjective or objective diagnostic tests were performed to evaluate the central auditory processing of the sample. However, it is worth mentioning that performing these procedures were outside the scope of the study proposal and the expected results, since the purpose of the present research was to perform a survey of the auditory manifestations of central auditory processing according to their symptoms, in order to determine the relevance of evaluating hearing-related cognitive functions in this population. The results of the research may be used to encourage further investigation on the subject of decodification of acoustic messages in patients with thyroid hypofunction. There is a need for new studies in the area that use standard verbal and non-verbal stimuli to evaluate hearing-related cognitive functions, and event-related cortical auditory evoked potentials (P300), which provide more support in differential diagnoses.

In conclusion, the results suggested that CH could be a risk factor for the development of CAPD considering the high frequency of lag symptoms in cognitive functions related to central auditory processing, especially in auditory attention, figure-background ability and auditory memory. Furthermore, according to the findings, the communicative, behavioral and school aspects may be impaired in individuals with CH, and the degree of severity of the lags was strongly associated with clinical and laboratory factors related to hormonal follow-up.
